# Neuroinflammatory Mechanisms of Adult Sepsis-Associated Encephalopathy: Implications for Blood–Brain Barrier Disruption and Oxidative Stress

**DOI:** 10.3390/diagnostics15070873

**Published:** 2025-03-30

**Authors:** Hao Liu, Ting Zhang, Lixiao Zhang, Yanjun Zhong

**Affiliations:** 1 Department of Critical Care Medicine, The Second Xiangya Hospital, Central South University, Changsha 410011, China; liuhao181@csu.edu.cn (H.L.); 238211186@csu.edu.cn (T.Z.); 2 Xiangya School of Medicine, Central South University, Changsha 410013, China; zhanglixiao@csu.edu.cn

**Keywords:** sepsis-related encephalopathy, neurotransmitters, signaling, blood–brain barrier, microcirculation, oxidative stress, mitochondrial disorders

## Abstract

Sepsis is a syndrome of life-threatening acute organ dysfunction caused by a dysregulated host response to infection. Sepsis-associated encephalopathy (SAE) refers to the diffuse brain dysfunction observed in sepsis cases, clinically characterized by a spectrum of neuropsychiatric manifestations ranging from delirium to coma. SAE is independently associated with increased short-term mortality and long-term neurological abnormalities, with currently no effective preventive or treatment strategies. The pathogenesis is intricate, involving disruptions in neurotransmitters, blood–brain barrier (BBB) breakdown, abnormal brain signal transmission, and oxidative stress, among others. These mechanisms interact or act in conjunction, contributing to the complexity of SAE. Scholars worldwide have made significant strides in understanding the pathogenesis of SAE, offering new perspectives for diagnosis and treatment. This review synthesizes recent mechanistic breakthroughs and clinical evidence to guide future research directions, particularly in targeting BBB restoration and oxidative stress.

## 1. Introduction

Sepsis, stemming from a dysregulated host response to infection, results in life-threatening multi-organ dysfunction [1]. It stands as a crucial cause of mortality in critically ill patients and is often described as one of the oldest and most elusive syndromes in medicine [2]. Additionally, it ranks among the leading causes of ICU admissions, accounting for 37.4% of hospitalized patients [3]. According to recent epidemiological research on sepsis in China, conducted in 2023, approximately 13.1% of mortality cases recorded between 2017 and 2019 were attributed to sepsis. Among these cases, 28.4% occurred during hospitalization, with an observed annual increase in in-hospital mortality rates. Globally, there were 48.9 million cases of sepsis worldwide in 2017 alone, of which 11 million people died as a result, accounting for 19.7% of all global deaths that year [1]. Clinical manifestations and symptoms are variable and usually associated with various comorbidities, such as altered mental status in the nervous system [4], acute kidney injury in the urinary system [5], and cardiomyopathy in the cardiovascular system [6]. Sepsis not only brings physical distress to patients but also imposes a significant economic burden on global healthcare systems. Studies indicate a sepsis incidence rate of up to 535 cases per 100,000 person-years in the United States, with a continuing upward trend [7]. Severe sepsis patients experience an annual increase of 8.2% in hospitalization and 5.6% in mortality rates [8], surpassing admissions for stroke and myocardial infarction [9]. This underscores sepsis as a major global cause of death and serious illness, drawing attention due to its high incidence, admission rates, and mortality rates.

The central nervous system is one of the most susceptible organs affected by sepsis, clinically manifested as sepsis-associated encephalopathy (SAE) [10]. It is the most common type of encephalopathy in the ICU and is characterized by diffuse cerebral dysfunction due to systemic reactions to the infection, and without clinical or laboratory evidence of direct intracranial infection or other types of encephalopathy (e.g., hepatic encephalopathy, etc.). This is manifested by altered consciousness (from delirium to coma), which may be accompanied by seizures (convulsive or nonconvulsive), or focal neurological signs, and may also be manifested by disturbances in consciousness that are incompatible with the degree of sedation, disturbances in the sleep–wake cycle, hallucinations, agitation, and other symptoms common in delirium [11]. The inflammatory immune response triggered by sepsis may not only cause the aforementioned symptoms but also lead to prolonged functional impairments [12]. Due to the lack of standardized diagnostic criteria, the prevalence of SAEs varies globally and between studies, ranging from 53% reported in a multicenter cohort of French ICUs [13] to 68% in a cohort of septic patients in the US databases MIMIC-IV and eICUs [14], but only 39% in a study from China [15]. However, research suggests a mortality rate of 49% in sepsis patients with altered mental status, compared to 26% in those without neurological symptoms [16]. The more severe the brain disease, the lower the survival rate. Moreover, up to 60% of sepsis survivors exhibit permanent cognitive deficits and memory loss [17], resulting in long-term cognitive dysfunction.

At present, the disease lacks clear diagnostic criteria, specific auxiliary examination, and therapeutic measures. In recent years, scholars have put a lot of effort into the pathogenesis of SAE, striving to alleviate the physical torment caused by SAE to patients, reduce the socio-economic pressure, and provide a new basis for the prevention, diagnosis, and treatment of SAE. In this paper, a review of the current research status of the pathogenesis of immune-mediated SAE is conducted by collecting and collating the literature.

**Figure 1:**
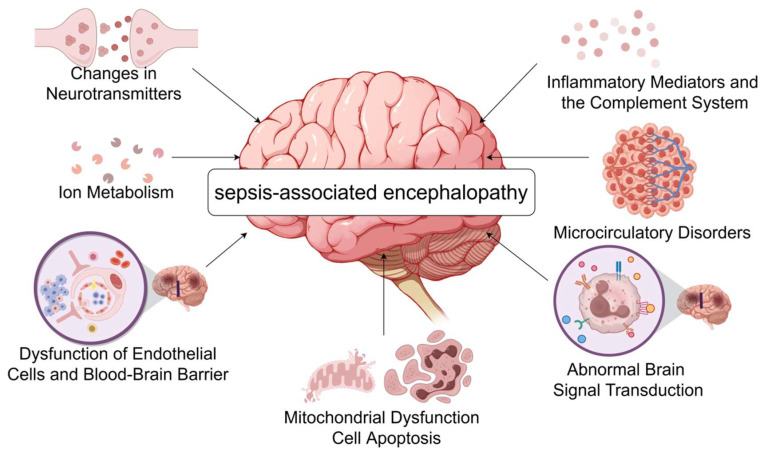
The pathogenesis of septic encephalopathy. During sepsis, inflammatory mediators such as IL-1β, TNF-α, and complement C5a reach the brain, causing an increase in BBB permeability and more harmful substances to enter the CNS, further aggravating neuroinflammation. At the same time, the release of large amounts of cytokines leads to neurotransmitter imbalance as well as abnormal brain signaling, causing patients to develop symptoms such as delirium. Systemic inflammation due to sepsis causes vasodilatation and extravasation of body fluids, and collective hypovolemia, resulting in inadequate blood supply to the brain and microcirculatory disturbances. The burst of neuroinflammation increases metabolic and energy demands, causing oxidative stress and mitochondrial dysfunction, ultimately leading to apoptosis. Changes in intracellular ion concentrations in neuronal cells appear to play an important role in septic encephalopathy (by Figdraw www.figdraw.com accessed on 6 July 2024).

The pathophysiology of sepsis-associated encephalopathy is complex and involves a variety of mechanisms that collectively lead to brain dysfunction and injury, the main ones currently accepted by scholars in various countries include the following (Figure 1).

## 2. Pathogenesis of SAE

### 2.1. Dysfunction of Endothelial Cells and Blood—Brain Barrier (BBB)

The blood–brain barrier is a crucial cellular barrier tightly controlling the microenvironment of the central nervous system to allow normal neuronal function. It is formed by endothelial cells of the capillary wall, astrocyte end-feet enveloping the capillaries, and pericytes embedded in the capillary basement membrane. Endothelial cells at the blood–brain barrier have unique characteristics compared to endothelial cells in other tissues, allowing them to tightly regulate the movement of ions, molecules, and cells between blood and the brain [18].

Cranial magnetic resonance imaging in patients with AE showed cerebral edema, suggesting the presence of BBB damage in patients with SAE [19], and indeed it has been shown that in the early stages of sepsis pathogenesis, nitric oxide derived from endothelial-type nitric oxide synthase in cerebrovascular endothelial cells has pro-oxidant effects that increase BBB permeability [20]. In addition, cytokines produced by immune cells during mobilization can cross the disrupted BBB and affect the central nervous system, resulting in increased vascular permeability, impaired vascular tone, platelet aggregation, and thrombosis, further exacerbating the body’s inflammatory response and leading to delirium [21,22], which may be attributed to the activation of endothelial cells by pro-inflammatory factors. This may be due to the activation of endothelial cells by pro-inflammatory factors such as TNF-α and IL-1β, the activation of which generates reactive oxygen species (ROS) that increase the permeability of the BBB, while the release of increased ROS damages the macromolecules of the blood–brain barrier, leading to mitochondrial dysfunction, the activation of matrix metalloproteinases, and ultimately damage to the blood–brain barrier [14].

In addition to the role of inflammatory factors, it has been found that the activation of mast cells can increase the sensitivity of mouse brain microvascular endothelial cells to LSP, and BBB disruption can be reduced to ameliorate cognitive dysfunction by inhibiting mast cells in the hippocampus of SAE mice [23], which also supports the above view. Meanwhile, complement C5a can cause BBB damage by activating neuroglia, releasing pro-inflammatory factors, and producing other harmful substances [24], and subsequent experiments have also shown that inhibition of complement C5a can reduce BBB damage in sepsis [25].

A loss of blood–brain barrier integrity plays a pivotal role in the pathophysiological process of SAE, and several factors can lead to the activation of endothelial cells and the alteration of the permeability of the BBB, which disrupts the relative isolation of the central nervous system, enabling a variety of neurotoxic substances that can directly damage the central nervous system and causing the relevant reactions, which ultimately leads to patients with cerebral dysfunction showing delirium and other clinical manifestations of SAE.

### 2.2. Oxidative Stress, Mitochondrial Dysfunction, and Cell Apoptosis

Although the brain constitutes only 2% of the body’s weight, it receives 15% of cardiac output, consumes 20% of total body oxygen, and utilizes 25% of total body glucose. The outbreak of neuroinflammation increases the demand for metabolism and energy, leading to oxidative stress reactions and mitochondrial dysfunction, ultimately resulting in cell apoptosis [26].

Oxidative stress is present in the early stages of sepsis and, on the one hand, oxidative stress is associated with endoplasmic reticulum stress and excessive endoplasmic reticulum stress is closely related to sepsis-induced neuronal damage [27], and on the other hand, oxidative stress leads to a decrease in the synthesis of intracellular ATP and an increase in the ratio between the activities of superoxide dismutase and catalase [28]. Meanwhile, several studies have shown that a large number of animal studies support the mitochondrial damage induced by oxidative stress and its role in the development of sepsis [29].

The onset of oxidative stress can be mediated by cytokines, reactive oxygen species (ROS), and nitric oxide (NO) [30]; for example, NO induces mitochondrial dysfunction by decreasing the affinity of cytochrome c oxidase for oxygen [31], which reduces ATP production, and the activation of NO, ROS, etc., also leads to mitochondrial dysfunction by inhibiting the electron transport chain complexes I and IV, which are involved in the onset and development of SAE. In addition, increased oxygen free radicals also cause oxidative stress, which further leads to oxidative damage in neurons [32]. Clinical studies have shown that the use of antioxidants can attenuate oxidative damage in the hippocampus induced by early sepsis [33].

Oxidative stress may also be associated with mitochondria-mediated apoptosis. Mitochondria-mediated apoptosis has been demonstrated in experimental sepsis and may be associated with a decrease in intracellular anti-apoptotic (e.g., bcl-2) factors and an increase in pro-apoptotic (e.g., bax) factors [34], and Comim et al. found that Caspase-3 was involved in partial apoptosis in the cell layer of the hippocampal dentate gyrus of septic rats [35], which led to long-term cognitive deficits. Gao et al. found that, by inhibiting the IL-17RA/Akt/ERK1/2 signaling pathway, oxidative stress and neuronal apoptosis could be alleviated, thereby ameliorating CLP-induced cognitive dysfunction in SAE rats [36].

**Figure 2:**
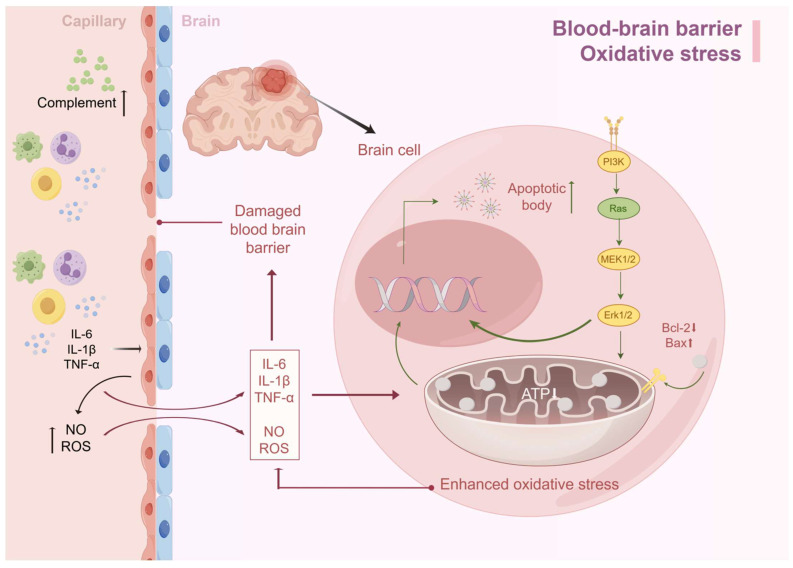
BBB destruction and oxidative stress lead to SAE. Excessive immune response during sepsis leads to the accumulation of cytokines such as IL-6, TNF-α, and IL-1β in serum, which synergistically increase BBB permeability by activating endothelial cells to produce reactive oxygen species and nitric oxide. Inflammatory factors infiltrating the brain parenchyma activate astrocytes and microglia, triggering a cascading inflammatory response that exacerbates BBB injury. Meanwhile, together with ROS, this induces oxidative stress, leading to mitochondrial dysfunction and insufficient ATP synthesis. This metabolic disorder triggers an imbalance between the reduction in anti-apoptotic factors and the increase in pro-apoptotic factors, which ultimately drives the apoptotic process (by Figdraw).

Interestingly, if we look at the damaging factors of oxidative stress, we can see that it has the same damaging factors as the BBB, such as ROS, NO, etc., and there is an interaction between the two. In the case of SAE, if there is damage to the BBB, inflammatory mediators such as TNF-α, IL-1β, IL-6, etc., can enter the brain through the damaged BBB and cause damage to the inner membrane of the mitochondria of the brain cells, which in turn causes mitochondrial dysfunction and oxidative stress, can enter the brain through the damaged BBB and cause damage to the inner membrane of the mitochondria of brain cells, resulting in mitochondrial dysfunction and oxidative stress. The decrease in ATP production caused by oxidative stress leads to insufficient energy synthesis, which in turn leads to neuronal edema and apoptosis, with acute manifestations such as delirium and long-term impaired consciousness (Figure 2).

### 2.3. Inflammatory Mediators and the Complement System

Sepsis leads to uncontrolled systemic inflammation, which may cause SAE when it reaches the brain. Meanwhile, inflammatory cytokines and the complement system are the ultimate common pathways in the pathophysiology of brain dysfunction in SAE. When the inflammatory response is excessively activated in sepsis patients, the large amounts of lipopolysaccharides and nitric oxide act on vascular endothelial cells, activating them to produce a significant amount of pro-inflammatory factors, including TNF-α, IL-1β, and IL-6, leading to BBB dysfunction and neurological inflammation [37]. Endotoxins can also activate the endothelial IκB-α/NF-κB pathway, increasing the production of IL-1β, TNF-α, and IL-6 [38]. These inflammatory factors enter the brain through diffusion, carrier proteins, receptor-mediated endocytosis, etc., continuously activating microglial cells, providing feedback to generate more inflammatory factors and reactive oxygen species, thereby increasing BBB permeability and neuronal apoptosis, progressing into SAE. Among these, TNF-α appears to be one of the most important inflammatory mediators in SAE. Inflammatory responses cause blood–brain barrier disruption, tissue edema, neutrophil infiltration, and cell apoptosis, all closely related to TNF-α/TNFR1 [39]. Increased expression of IL-1 can stimulate the production of prostaglandin E2 in brain vascular endothelial cells, acting on the hypothalamus, brainstem, and limbic system, ultimately leading to cognitive dysfunction [40]. Abnormal activation of the inflammatory body NLRP3 is associated with many inflammatory diseases, and sepsis-triggered typical inflammasome depends on the NLRP3/caspase-1 pathway to mature and secrete IL-1β, which may lead to the reduced generation of new neurons [41]. Complement activation is an early observable phenomenon [42]. Researchers have found that endotoxins can increase C5a in brain endothelial cells and microglial cells [43], and the inhibition of complement C5a or the inhibition of its receptor can also reduce BBB damage in sepsis [25,44]. Meanwhile, the upregulation of complement C3 also causes changes in the BBB [45], increasing its permeability to enable the invasion of inflammatory mediators into the brain, leading to edema, cell death, or neuronal apoptosis. It has been hypothesized in the literature that microglia activated by C3 and C3aR may induce the loss of inhibitory synapse-associated proteins by disrupting GABAergic synaptic transmission, ultimately exacerbating cognitive impairment [46]. However, this hypothesis has not been tested.

### 2.4. Changes in Neurotransmitters

Neurotransmitters are chemical substances that transmit information between neurons or between neurons and effector cells such as muscle cells and gland cells. They play a crucial role in transmitting nerve impulses and regulating the excitability and inhibitory functions of neurons when the brain is functioning. Alterations in neurotransmitters such as acetylcholine, γ-aminobutyric acid (GABA), and norepinephrine in the patient’s body influence the occurrence and maintenance of SAE [47]. The massive release of cytokines during the sepsis process may lead to disturbances in neural transmission. Some scholars have found that sepsis is related to inflammation and metabolism, leading to changes in brain neurotransmission. It has been suggested that cytokines activate glial cells in the brain during the sepsis process, causing their immune defense function to shift to cytotoxicity. This, in turn, releases inflammatory mediators in the central nervous system, leading to abnormal neural function and the development of SAE [45,48].

In a prospective cohort study, academics demonstrated that altered acetylcholinesterase activity during critical illness leads to acute brain dysfunction in patients [49]. Low cholinergic activity is thought to be an important cause of cognitive dysfunction during delirium [50], which not only triggers the release of various pro-inflammatory cytokines, but also activates microglia and amplify the neuroinflammatory response [51], while cholinergic transmitters shorten the duration of delirium, and more severe and long-lasting alterations in consciousness occur if the patient has an impaired cholinergic function or a history of anticholinergic medication administration [48]. This is supported by the fact that cholinergic drugs have been found to reverse memory impairment and cognitive deficits in sepsis-surviving rats in recent years [52]. However, a clinical study showed that cholinesterase inhibitors increased delirium duration and morbidity and mortality in the treatment of critically ill patients [53]. Although the results of current studies on the cholinergic system are different, we cannot completely rule out the role of a cholinergic neurotransmitter imbalance in the onset and development of SAE. At the same time, the possibility that it may interact with other neurotransmitters in the body and thus contribute to SAE cannot be ignored. These questions need to be investigated further.

Some amino acids that can be converted into neurotransmitters may also be correlated with SAE. For example, tryptophan is the precursor of serotonin, glutamate is converted to GABA, and the synthesis of dopamine and norepinephrine requires phenylalanine and tyrosine. Changes in the levels of these amino acids affect neurotransmitter synthesis. Analysis of cerebrospinal fluid protein in sepsis patients has shown significant abnormalities in the concentrations of essential amino acids needed for synthesizing the above neurotransmitters. Pandharipande et al. demonstrated that extremely low levels of tryptophan and abnormal levels of tyrosine are associated with an increased risk of developing delirium, although the specific mechanisms are not fully understood, indicating that changes in amino acids may play an important role in the pathogenesis of ICU delirium [54]. At the same time, research has found that glial cells in sepsis patients are activated and release a large amount of glutamate. The use of glutamate contributes to apoptotic neuronal death, indirectly suggesting the involvement of glutamate in the formation of sepsis-associated brain diseases [55]. Berg et al. found a decrease in the ratio of branched-chain amino acids to aromatic amino acids in the plasma of LPS-injected volunteers, mainly due to a decrease in valine and isoleucine, and an increase in phenylalanine. Phenylalanine can be converted into a pseudo-neurotransmitter, inducing brain diseases, and emphasizing the important role of phenylalanine in the pathophysiology of SAE [56]. In addition, the excessive release or reduced clearance of neurotransmitters such as glutamate and glutathione can lead to increased neuronal excitability, affecting brain function [57]. The disruption of tryptophan metabolism is also involved in the pathophysiological process of cognitive impairment induced by sepsis [58].

Adrenaline also affects the occurrence and development of SAE. Shehabi et al. found that in septic patients, the use of adrenergic drugs such as dexmedetomidine as the sole sedative for critically ill patients in the ICU not only does not reduce mortality but also leads to more adverse reactions such as delirium [59]. However, clinical studies by Pandharipande et al. suggest that, compared to midazolam or lorazepam, dexmedetomidine has fewer cognitive impairments and better outcomes in septic patients [60]. The conclusions of the two experimental groups happen to be contradictory, requiring further research, but they can explain the impact of adrenaline on SAE.

### 2.5. Abnormal Brain Signal Transduction

During sepsis, various factors may influence the brain’s signal transduction. Research indicates that during sepsis, the brain signals rely on two main pathways—the vagus nerve and circumventricular organs (CVOs)—with peripheral cortisol playing a feedback regulatory role [61]. Massive pro-inflammatory cytokines produced by microbial invasion reach crucial structures in the brain through CVOs and damaged areas of the blood–brain barrier (BBB) or actively traverse the BBB through specific carriers. Simultaneously, the axon cell receptors of the vagus nerve are activated in the periphery, connecting the vagus nerve nucleus to various autonomic nerve nuclei such as the adrenal axis and the paraventricular nucleus regulating anti-diuretic hormone secretion, causing various hormone secretions and abnormal reflex regulation [62]. Studies have confirmed the early activation of microglial cells in septic mouse models, possibly one of the earliest changes observed in SAE [63]. These activated neuroglial cells acquire neurotoxic characteristics [64] and release a large number of inflammatory and anti-inflammatory factors, such as nitric oxide, glutamate, and prostaglandins, which act on brain neurons leading to disruptions in neural transmission and secretion; for example, an excess of nitric oxide and complement anaphylatoxin C5a may cause DNA damage to hippocampal and hypothalamic neurons [62], resulting in cognitive dysfunction. Scholars have also found that these pro-inflammatory mediators released in the central nervous system during the onset of sepsis can lead to the loss of neurons in vulnerable areas of the brain, including the hippocampus [34,65]. These changes are closely related to manifestations of SAE, such as delirium.

### 2.6. Cerebral Perfusion and Microcirculatory Disorders in the Brain

Abnormal cerebral perfusion refers to hemodynamic abnormalities in the brain, a typical manifestation of cerebrovascular diseases. Adequate cerebral perfusion and microcirculatory function are crucial for maintaining normal brain function. Increasing evidence supports changes in cerebral perfusion and microcirculation during sepsis. Post-mortem analyses of sepsis patients using MRI, as well as animal experiments, confirm the presence of ischemic and hemorrhagic lesions in macroscopic and microscopic regions [66,67]. These findings suggest that these changes may play a significant role in the pathogenesis of SAE. Clinical studies confirm that systemic circulatory disorders in SAE patients lead to heart failure, causing hypotension, resulting in changes in cerebral microcirculation and ultimately reducing cerebral perfusion [68,69]. Under normal physiological conditions, the brain can maintain a constant cerebral blood flow through autoregulation. However, in approximately 50% of SAE patients, the brain loses its autoregulation function, making it more susceptible to changes in mean arterial pressure and causing insufficient cerebral perfusion [70,71]. Additionally, sepsis-induced systemic inflammatory reactions can lead to vasodilation and fluid extravasation, slowing blood flow in vessels and causing inadequate cerebral blood supply, resulting in brain dysfunction and potentially contributing to the occurrence of SAE [72]. Scholars have also found a correlation between septic shock in experimental sheep and a decrease in overall cerebral vascular density and the proportion of small vessels during full perfusion. They directly observed microcirculatory disorders in the sheep’s brains using sidestream dark-field microscopy [73]. The breakdown of the BBB in sepsis patients, as mentioned earlier, leads to brain edema, which, due to its compressive effect, also causes a decrease in cerebral perfusion. In SAE patients, cerebral blood flow is slowed, and vascular resistance is increased. The hemodynamic instability caused by sepsis, the release of numerous cytokines, and changes in the synthesis of nitric oxide from endothelial nitric oxide synthase in cerebral blood vessel endothelial cells lead to impaired cerebral vascular contractile function, further causing microcirculatory disorders in the brain [74]. Additionally, the aforementioned factors affect coagulation function, leading to capillary thrombosis and subsequent neuronal hypoxia and apoptosis. All these findings indicate that the reduction in cerebral perfusion and microcirculatory disorders are important causes of SAE.

### 2.7. Ion Metabolism

Some researchers propose that the increase in intracellular calcium ion concentration is related to sepsis-induced brain damage. They have also demonstrated that the level of calcium ions in the hippocampal cells of septic rats is elevated, and changes in calcium ion concentration can affect neurotransmitter generation and transmission, leading to damage to the learning, memory, and cognitive functions [75]. Inflammatory reactions can inhibit the absorption of intestinal iron, decrease the transport and utilization of iron in the blood, causing abnormal iron metabolism in the body, directly affecting the neurological function of sepsis patients, and subsequently leading to the occurrence of SAE. Furthermore, a prospective study found an association between systemic iron homeostasis changes and the prognosis of sepsis patients [76].

## 3. Summary and Discussion

Sepsis is a common disease with potential risks of mortality and a high economic healthcare burden. Brain dysfunction is common in sepsis and is a significant risk factor for mortality but is often overlooked because SAE can occur at any stage of sepsis, and effective preventive and treatment strategies have yet to be discovered. Studies have shown that in intensive care units, SAE patients account for about 70% of all sepsis patients, and the long-term quality of life for survivors is also poor [72].

Nevertheless, it is fortunate that an increasing number of scholars recognize the importance of thoroughly researching SAE and have gained a further understanding of its pathogenesis. There is ample evidence supporting that pathological physiological processes such as oxidative stress, blood–brain barrier dysfunction, and mitochondrial dysfunction occur in the brains of sepsis patients, mediating the occurrence of SAE, and have gained support from scholars worldwide. However, the relationship between these changes and cognitive dysfunction has not been well studied. At the same time, in sepsis as a systemic syndrome, various factors are not independent of each other, but influence and collaborate to jointly mediate the occurrence of SAE; for example, inflammatory mediators activate endothelial cells leading to the dysfunction of the BBB, and activated endothelial cells can generate new inflammatory mediators and complement to continue to aggravate the increase in the permeability of the BBB.

A recent study found that the gut–brain axis plays a pivotal role in the development of SAE. Dysbiosis of intestinal flora in sepsis patients can induce delirium through various pathways [77]. The abundance of probiotics such as Prevotella spp. in the early intestinal tract of septic rats was significantly reduced, and the abundance of opportunistic pathogens such as Helicobacter spp. was significantly increased [78]. Simvastatin sodium and metformin reduce septic rat mortality by improving intestinal flora and metabolites in septic rats [79,80]. Exogenous supplementation with phosphatidylserine modulates the gut microbiome, maintains intestinal integrity, and improves brain pathology in mice surviving sepsis [81]. These all indicate that the pathogenesis of SAE currently explored is just the tip of the iceberg, and there is much unknown that needs further exploration.

One of the reasons for the high incidence and mortality rates of SAE is the lack of unified diagnostic criteria, especially the lack of objective biochemical and imaging indicators, leading to delayed diagnosis. In addition, the treatment of SAE depends on treating sepsis (early sepsis treatment includes appropriate antibiotics, restoring sufficient perfusion of tissues and organs, and timely control of the source of infection), correcting possible neurotoxic factors, and symptomatic supportive treatment for seizures, delirium, and coma, etc. There is a lack of effective prevention and treatment methods [82,83]. Therefore, while exploring its pathogenesis, scholars should also focus on uncovering characteristic factors to guide the early diagnosis of SAE, such as neuron-specific enolase (NSE) and neurofilament light (NfL), as well as biomarkers associated with glial damage.

On the therapeutic side, today’s targeted drugs have achieved remarkable results in tumor therapy, so we should start from these mechanisms to find effective drug targets or potential therapies for sepsis-associated encephalopathy (SAE). For example, recent studies have shown that the activation of GSDMD in brain endothelial cells underlies the disruption of the blood–brain barrier by lipopolysaccharides [84], which provides new ideas for the treatment of central nervous system diseases associated with blood–brain barrier damage. In addition, Chinese herbs have performed well in the fight against the new crown epidemic, and scientists are studying the mechanism of action of their active ingredients in depth. We can consider combining Chinese herbs with the pathogenesis of SAE to explore their therapeutic potential. For example, berberine can alleviate the neuroinflammation of SAE through the NFκB/LCN2 pathway and reduce brain damage and inflammatory infiltration [85]. In addition, new therapies such as intestinal flora transplantation, IL-6 receptor blockade therapy, and nanomedicine research, as well as mesenchymal stem cells to promote blood–brain barrier repair, are being developed to provide an early solution to the challenges of SAE treatment. Other emerging therapies such as intestinal flora transplantation, immunotherapy, and nanomedicines are constantly being updated to strive for an early resolution of the SAE treatment challenge [86,87,88,89].

In summary, the pathogenesis of SAE is highly complex, and various inflammatory and non-inflammatory processes affect its occurrence and maintenance. It is worthwhile for us to further study and explore to obtain more effective information for rational application in diagnosis and treatment, bringing good news to the vast number of patients.

## Data Availability

No data were used for the research described in the article.

## Abbreviations

SAE: Sepsis-associated encephalopathy; ICU: intensive care unit; GABA: γ-aminobutyric acid; LPS: lipopolysaccharide; CVOs: circumventricular organs; BBB: blood–brain barrier; TNF-α:tumor necrosis factor-α; IL-1β: Interleukin-1β; IL-6: Interleukin-6; ROS: reactive oxygen species; CNS: central nervous system; ER: endoplasmic reticulum; GSDMD: gastrin D; HMGB1: high mobility group box 1 protein; COVID-19: Corona Virus Disease 2019; NO: nitric oxide; bax: recombinant Bcl2-associated X; bcl-2: B-cell lymphoma-2.
